# Effect of Vitamin D3 Supplementation on Insulin Sensitivity in Prediabetes With Hypovitaminosis D: A Randomized Placebo-Controlled Trial

**DOI:** 10.7759/cureus.12009

**Published:** 2020-12-10

**Authors:** Munibuddin M Ahmed, Urjita S Zingade, Khaled M Badaam

**Affiliations:** 1 Physiology, Government Medical College, Aurangabad, IND; 2 Physiology, Rajarshi Chhatrapati Shahu Maharaj Government Medical College, Kolhapur, IND

**Keywords:** oral glucose insulin sensitivity (ogis) index, vitamin d deficiency, insulin resistance, glucose, type b insulin resistance, glucose metabolism, impaired glucose tolerance, 25 (oh) vitamin d, vitamin d

## Abstract

Introduction

The interplay of vitamin D and glucose metabolism is an area of ongoing research. The need for vitamin D supplementation trials in individuals with prediabetes and hypovitaminosis D has been stressed by earlier research studies. The objective of this study was to assess the effect of vitamin D3 supplementation on oral glucose insulin sensitivity (OGIS) index in patients with prediabetes and hypovitaminosis D.

Methods

We enrolled 120 individuals with prediabetes (ADA definition) and hypovitaminosis D (vitamin D < 30 ng/mL) and randomized them into the vitamin D supplementation (60,000 IU weekly) group and the placebo group. Primary outcome measure (i.e., 2-hour OGIS index) and secondary outcome measures (i.e., fasting and postprandial blood glucose, glycosylated hemoglobin, body mass index, and insulin sensitivity indices, i.e., quantitative insulin sensitivity check index [QUICKI] and homeostatic model assessment for insulin resistance [HOMA-IR]) were analyzed for change with the 12 weeks of intervention.

Results

A total of 52 subjects in the vitamin D group and 49 in the placebo group completed the study. Serum vitamin D levels (10.11 ± 2.73 to 52.2 ± 13.14 ng/mL; p < 0.0001) and OGIS index (376.4 ± 39.7 to 391.7 ± 40.7 mL/min/m^2^; p = 0.011) increased significantly on per-protocol analysis in the vitamin D group. There was no significant change observed in vitamin D levels and OGIS index in the placebo group. Between-group comparison showed a rise in OGIS index (15.3 ± 47.1 mL/min/m^2^) in the vitamin D group and decrease in OGIS index (-10.4 ± 44.7 mL/min/m^2^) in the placebo group, and the difference was statistically significant (p = 0.0029). The inter-group comparison showed relative fall in fasting glucose levels in the vitamin D group, with no significant change observed in the other secondary outcome measures.

Conclusions

The correction of hypovitaminosis D in subjects with prediabetes led to improved insulin sensitivity as assessed by OGIS index at 120 minutes, signifying the role of vitamin D in glucose homeostasis.

## Introduction

A recent Indian Council of Medical Research-India diabetes (ICMR-INDIAB) study has estimated a population prevalence of prediabetes in India to be 24.7%, i.e., almost a quarter of adult population belongs to a high-risk group that can progress to diabetes mellitus in the near future [1]. Also, the prevalence of hypovitaminosis D has reached epidemic proportions in spite of the abundant sunshine in the region [2]. Studies in animals and human cohort studies have found an association of vitamin D with insulin secretion and action. An inverse association of vitamin D levels with insulin resistance has been found by observational studies [3,4]. However, supplementation trials with vitamin D in subjects with deranged glucose metabolism have reported inconsistent results. There has been an interest in studying the impact of vitamin D supplementation on glycaemic indices in subjects with prediabetes and hypovitaminosis D. Oral glucose tolerance test (OGTT) based insulin sensitivity test, i.e., oral glucose insulin sensitivity (OGIS) index at 120 minutes, is a simple surrogate marker related to pathophysiology of diabetes that can be used for research studies in large subjects [5]. With this background, this study was undertaken to evaluate the effect of oral vitamin D3 supplementation on OGIS and glycemic status in the subjects with prediabetes and hypovitaminosis D.

## Materials and methods

This parallel-group double-blind randomized placebo-controlled trial was conducted at Government Medical College in Marathwada region of Maharashtra. The hypothesis was that vitamin D3 supplementation improves OGIS index in prediabetes subjects with hypovitaminosis D. Sample size was calculated as 49 subjects per group for one-tailed hypothesis testing (α error at 5% for 80% power of study) to compare the change in OGIS values between the groups. The mean OGIS index and anticipated improvement in OGIS from the available literature were used for the calculation [6,7]. Due to possible attrition, the sample size was increased to 60 in each group. Approval was obtained prior to data collection from the Institutional Ethics Committee. Informed written consent was obtained from all the participants in the language known to them. Trial registration was done with the Clinical Trial Registry of India (CTRI/2012/12/003221) and the World Health Organization registry network (URN: U1111-1134-6536). This trial was conducted in accordance with the principles of the Helsinki declaration.

Participants and eligibility criteria

Eligible participants were adults aged >25 years with prediabetes and hypovitaminosis D. Prediabetes was defined as per the American Diabetes Association (ADA) criteria, i.e., fasting plasma glucose of 100 to 125 mg/dL or 2-hour glucose concentration of 140 to 199 mg/dL after 75-g oral glucose, or glycosylated hemoglobin (HbA1c) of 5.7% to 6.4% [8]. Hypovitaminosis D was defined as serum 25(OH)D < 30 ng/mL [9] as per the Institute of Medicine guidelines. All known cases of diabetes, prediabetes patients on pharmacotherapy, subjects using supplements that contained vitamin D, and known cases of hypercalcemia, hyperparathyroidism, nephrolithiasis, chronic kidney disease, or other major illness were excluded. Participants were recruited from the medical college, from private hospitals, and through medical camp and direct contacts.

Outcome measures

Primary outcome measure was OGIS index at 120 minutes. Secondary outcome measures were fasting blood glucose, post-prandial blood glucose, HbA1c, body mass index (BMI), and insulin sensitivity indices (quantitative insulin sensitivity check index [QUICKI] and homeostatic model assessment for insulin resistance [HOMA-IR]).

Screening

History taking, general examination, systemic examination, and necessary investigations were performed in the subjects willing to participate in the study. Height was measured by a portable stadiometer marked up to 200 cm. Weight was measured by a portable weighing machine, with a maximum capacity of 180 kg. BMI was calculated as weight in kilograms divided by the square of height in meters. Screening of 157 high-risk subjects was done by the physician based on ADA reports (having BMI > 25 kg/m^2^, physical inactivity, first degree relative of a diabetic) [10,11]. All the subjects were investigated for 2-hour OGTT (0, 90, and 120 minutes), HbA1c, and vitamin D levels. A total of 37 subjects were excluded as 29 were normoglycemic and eight were having normal vitamin D levels. The enrolled 120 subjects were allotted serial numbers.

Sample collection and its evaluation

After an overnight 12-hour fast, under aseptic precautions, 4 mL of venous blood was collected in a syringe for the measurement of HbA1c, plasma glucose, serum insulin, and serum 25(OH)D. Two-hour OGTT with a solution containing 75-gm glucose was performed. Blood samples were again drawn after 90 and 120 minutes for plasma glucose and serum insulin measurement. Plasma glucose was measured at 0, 90, and 120 minutes, and the serum insulin was measured at 0 and 90 minutes. Plasma glucose (mg/dL) was estimated using the glucose oxidase peroxidase (GOD-POD) method [12]. Serum insulin (µU/mL) and 25(OH)D (ng/mL) were estimated by electrochemiluminescence immunoassay (ECLIA®; Elecsys, Roche, Mannheim, Germany). HbA1c (%) was measured using ion-exchange HPLC (D-10™, Bio-Rad, Hercules, CA, USA). OGIS index (mL/min/m^2^) [13] was calculated from Excel spreadsheet available at http://webmet.pd.cnr.it/ogis/. QUICKI was evaluated by the following formula using the calculator available at https://sasl.unibas.ch/11calculators-QUICKI.php: 1 / [(log (fasting insulin in µU/mL) + log (fasting glucose in mg/dL)] [14,15]. HOMA-IR was calculated according to the following formula using Excel spreadsheet available at https://www.dtu.ox.ac.uk/homacalculator/: fasting insulin (µU/L) x fasting glucose (nmol/L)/22.5 [16].

Randomization and intervention

Eligible participants were randomly assigned in a 1:1 ratio to receive oral vitamin D soft gel (60,000 IU cholecalciferol) or matching placebo once a week (after breakfast) for 12 weeks. Randomization was performed using a computer-generated random-number sequence by the pharmacist. Allocation concealment was done by opaque containers. Participants were advised to maintain their usual diet and physical activity and to avoid taking any supplements containing vitamin D during the study. Compliance with consumption of medicine or placebo was assessed by phone follow-up and returning the container. Twelve weeks after supplementation, participants came to the center for their repeat testing of the outcome parameters. Out of the 120 subjects enrolled, 101 subjects reported for the post-intervention follow-up visit. The entire program was offered free of charge. The investigator, study, subjects, and statistician were blinded regarding the subject allocation.

Statistical analysis

The data were compiled and expressed as mean ± SD. The statistical analysis was performed using Microsoft Excel and online calculators available at http://www.graphpad.com/quickcalcs and https://www.socscistatistics.com. A p-value of <0.05 was considered as statistically significant. All the variables were measured in both the groups before and after the supplementation of placebo or vitamin D. Final analysis was performed after 12 weeks of supplementation. All the variables were assessed according to per-protocol analysis (n = 52 + 49 = 101), whereas serum 25(OH)D and the primary objective, i.e., OGIS index, were also assessed as per intention-to-treat (ITT) analysis (n = 60 + 60 = 120) with the baseline observations carried forward (BOCF). Test for normality of data (Shapiro-Wilk test) was applied and based on that parametric (paired and unpaired t-test) or nonparametric tests (Wilcoxon signed-rank test and Mann-Whitney U test) were used for analysis.

## Results

**Figure 1:**
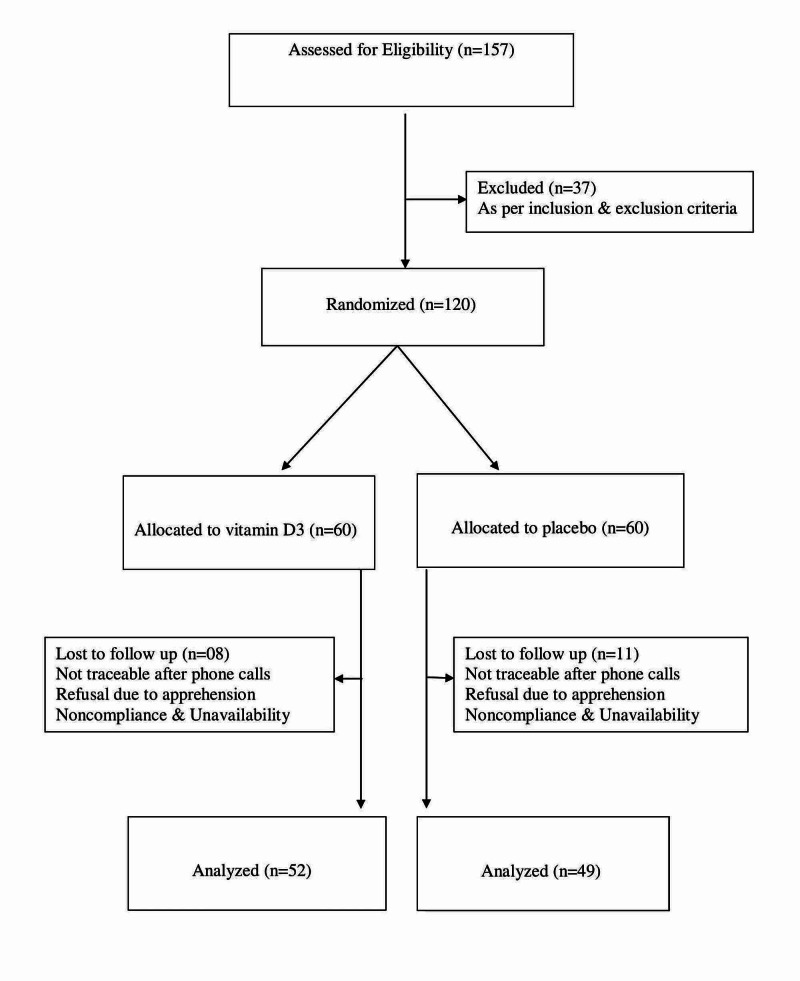
Flow chart of the study participants

A total 101 subjects completed the study: 52 from the vitamin D group and 49 from the placebo group. Figure 1 shows the flow chart of the study progression. Mean age of the subjects was 41.1 ± 8 years in the vitamin D group and 41.6 ± 7 years in the placebo group (Table 1). After the intervention, on per-protocol analysis and within-group comparison, there was a significant improvement in vitamin D levels (10.1 ± 2.7 to 52.2 ± 13.1 ng/mL; p < 0.001) in the vitamin D supplementation group, with no significant change in the placebo group. All the subjects in the vitamin D supplementation group attained vitamin D sufficiency. Insulin sensitivity as evaluated by OGIS index at 2 hours also increased significantly in the vitamin D supplementation group from 376.4 ± 39.7 to 391.7 ± 40.7 mL/min/m^2^ (p = 0.011), whereas there was a fall in the OGIS index in the placebo group, although the difference was not statistically significant (Table 2). On ITT analysis, similar results were observed on within-group comparison, with a significant rise in vitamin D levels and OGIS index in the vitamin D supplementation group (Table 3). Between-group comparison on per-protocol analysis showed an improvement in OGIS index (+15.3 ± 47.1) in the vitamin D group and decrease in OGIS index (-10.4 ± 44.7) in the placebo group, and difference was statistically significant (p = 0.0029). Between-group comparison with ITT analysis showed a statistically significant improvement in the OGIS index in the vitamin D supplementation group (Table 4). Secondary outcome measures were analyzed with per-protocol method, and there was a significant change observed only in fasting glucose (between-group comparison). No significant change was observed in BMI, HbA1c, fasting insulin levels, post-meal glucose and insulin levels, and insulin sensitivity index QUICKI and HOMA-IR between the groups (Table 5).

**Table 1:** Baseline characteristics of the study subjects SD, standard deviation

Variables	Vitamin D Group (Mean ± SD)	Placebo Group (Mean ± SD)
	(n = 52)	(n = 49)
Age (years)	41.1 ± 8	41.6 ± 7
Height (meters)	1.6 ± 0.1	1.6 ± 0.1
Weight (kg)	68.5 ± 11.9	70.7 ± 10.7
Body mass index (kg/m^2^)	26 ± 3.1	26.3 ± 2.7

**Table 2:** Per-protocol analysis of serum 25 (OH)D and OGIS index *Highly significant; **Significant. SD, standard deviation; CI, confidence interval; OGIS, oral glucose insulin sensitivity

Variable	Vitamin D Group (Mean ± SD), n = 52				Placebo Group (Mean ± SD), n = 49			
	Pre-Vitamin D Supplementation	Post-Vitamin D Supplementation	Mean Difference [95% CI]	p-Value	Pre-Placebo	Post-Placebo	Mean Difference [95% CI]	p-Value
Serum 25(OH)D (ng/mL)	10.1 ± 2.7	52.2 ± 13.1	42.1 [38.5 to 45.7]	<0.001*	13.7 ± 5.7	12.5 ± 4.8	-1.2 [-3.1 to 0.8]	0.153
OGIS index (mL/min/m^2^)	376.4 ± 39.7	391.7 ± 40.7	15.3 [2.5 to 28.1]	0.011**	366.4 ± 35.7	355.9 ± 30.1	-10.4 [-22.9 to 2.1]	0.054

**Table 3:** Intention-to-treat analysis of serum 25(OH)D and OGIS index *Highly significant; **Significant. SD, standard deviation; CI, confidence interval; BOCF, baseline observations carried forward; OGIS, oral glucose insulin sensitivity

Variable	Vitamin D Group (Mean ± SD), n = 60				Placebo Group (Mean ± SD), n = 60			
	Pre-Vitamin D Supplementation	Post-Vitamin D Supplementation (BOCF)	Mean Difference [95% CI]	p-Value	Pre-Placebo	Post-Placebo (BOCF)	Mean Difference [95% CI]	p-Value
Serum 25(OH)D (ng/mL)	10.1 ± 2.6	46.6 ± 19	36.5 [31.7 to 41.3]	<0.001*	13.8 ± 5.9	12.8 ± 5.3	-1 [-2.6 to 0.6]	0.307
OGIS index (mL/min/m^2^)	376 ± 39	389.3 ± 40.4	13.3 [2.1 to 24.5]	0.011**	370.3 ± 37	361.7 ± 33.6	-8.5 [-18.8 to 1.8]	0.054

**Table 4:** Comparison of change observed in serum 25(OH)D levels and OGIS index between groups *Highly significant; **Significant. SD, standard deviation; OGIS, oral glucose insulin sensitivity

Variable	Per-Protocol Analysis			Intention-to-Treat Analysis		
	Change in Vitamin D Group (Mean ± SD), n = 52	Change in Placebo Group (Mean ± SD), n = 49	p-Value	Change in Vitamin D Group (Mean ± SD), n = 60	Change in Placebo Group (Mean ± SD), n = 60	p-Value
Serum 25(OH)D (ng/mL)	42.1 ± 13.2	-1.2 ± 7	<0.001*	36.5 ± 19	-1 ± 6.3	<0.001*
OGIS index (mL/min/m^2^)	15.3 ± 47.1	-10.4 ± 44.7	0.002**	13.3 ± 44.1	-8.5 ± 40.6	0.002**

**Table 5:** Comparison of secondary outcome variables before and after supplementation *Statistically significant. SD, standard deviation; HbA1c, glycosylated hemoglobin; QUICKI, quantitative insulin sensitivity check index; HOMA-IR homeostatic model assessment for insulin resistance

Variables (Mean ± SD)		Pre-Vitamin D supplementation	Post-Vitamin D supplementation	p-Value	Pre-Placebo Supplementation	Post-Placebo Supplementation	p-Value	Difference with Vitamin D Supplementation	Difference with Placebo Supplementation	p-Value
Body mass index (kg/m^2^)		26 ± 3.1	26 ± 3	0.315	26.3 ± 2.7	26.2 ± 2.8	0.146	0.04 ± 0.46	-0.09 ± 0.68	0.121
HbA1c (%)		5.8 ± 0.4	5.7 ±0.4	0.146	5.8 ± 0.4	5.8 ± 0.4	0.206	-0.07 ± 0.5	0.05 ± 0.3	0.079
Glucose (mg/dl)	0 min	106.4 ± 10.8	103.6 ± 11.7	0.078	110.5 ± 10.4	114.2 ± 9.2	0.053	-2.8 ± 14.2	3.7 ± 14.5	0.018*
	90 min	154.6 ± 24.7	156.7 ± 21.2	0.197	158.2 ± 20.7	160.8 ± 17.6	0.284	2.2 ± 19	2.7 ± 27.4	0.459
	120 min	139.3 ± 18.5	137.5 ± 17.5	0.22	141.2 ± 14.7	144.6 ± 13.9	0.178	-1.7 ± 15.9	3.4 ± 20.7	0.083
Insulin (µU/mL)	0 min	7.8 ± 3.6	7.2 ± 3	0.197	6.9 ± 3.6	7 ± 3.4	0.452	-0.6 ± 4.7	0.1 ± 2.6	0.251
	90 min	51.4 ± 20	46.5 ± 20	0.203	48.5 ± 23.1	47.7 ± 17.4	0.456	-4.9 ± 28.6	-0.9 ± 26.9	0.301
QUICKI		0.35 ± 0.04	0.35 ± 0.02	0.274	0.36 ± 0.04	0.35 ± 0.03	0.178	00 ± 0.04	-0.01 ± 0.03	0.079
HOMA-IR		2 ± 1	1.9 ± 0.8	0.125	1.9 ± 1	2 ± 0.9	0.211	-0.2 ± 1.2	0.1 ± 0.7	0.091

## Discussion

All the study participants became vitamin D sufficient (25(OH)D > 30 ng/mL) after supplementation with 60,000 IU of vitamin D3 soft gels weekly for 12 weeks. There was no significant change in vitamin D levels in the placebo group. There were no subjects with more than 100 ng/mL values of 25-hydroxy vitamin D in our study group. Normalization of vitamin D levels with high-dose vitamin D supplements for a short period has been reported earlier [17].

The study results showed that there was an improvement in insulin sensitivity (OGIS index at 120 minutes) after correction of hypovitaminosis D. The improvement in OGIS index was around 4% in the vitamin D group, which is not large enough. Still, it was in contrast to a slight fall (3%) in the OGIS index observed in the placebo group. The between-group comparison reflects a 7% statistically significant difference between the groups. Among the secondary outcome measures, fasting glucose showed improvement. In contrast, there was no significant change in HbA1c, BMI, post-prandial glucose levels, and insulin sensitivity measures based on fasting glucose and insulin values, i.e., HOMA-IR and QUICKI.

OGIS index included an overall assessment of insulin sensitivity and involved fasting and post-prandial status of glucose and insulin levels; therefore, change in either or both fasting and post-prandial insulin sensitivity impact the value of OGIS. Thus, even with moderate changes in HOMA-IR and QUICKI, which assess only the fasting status values of glucose and insulin, there can be a significant improvement in OGIS values. OGIS index has been reported to be a better surrogate marker of insulin sensitivity in comparison with other indices such as Matsuda index, QUICKI, and HOMA-IR, especially in subjects with prediabetes [5,18].

The presence of vitamin D response element has been demonstrated on the human insulin receptor gene promoter, and 1,25 dihydroxy D3 has been shown to induce activation of the insulin receptor gene, potentiating the insulin response [19,20]. Insulin resistance in skeletal muscle mediated by forkhead box O1 (FOXO1), a critical negative regulator of insulin signaling, has been found to be linked to vitamin D [21]. Gröber and Holick reviewed the role of vitamin D in optimizing glucose metabolism and commented that vitamin D increases insulin sensitivity, thereby improving glucose tolerance [22]. A recent systematic review and meta-analysis by He et al. explored the vitamin D supplementation effect in nondiabetics. They found that fasting glucose levels were not changed by vitamin D supplementation in normoglycemic people and in individuals with normal vitamin D levels. Still, on subgroup analysis, they found that there was a fall in fasting glucose observed in subjects with prediabetes or low vitamin D. It was observed that in vitamin D deficient subjects (<20 ng/mL), vitamin D repletion may prevent progression to diabetes [23]. The range of vitamin D deficiency spectrum in which there is a maximal response to correction of vitamin D deficiency is reported to be 16-36 ng/mL. This range is speculated to have two to seven times more interaction of vitamin D3 with various metabolic parameters [24,25]. An improvement in the OGIS index by the supplementation of 50,000 IU vitamin D per week for one year in the subjects with prediabetes and hypovitaminosis D has been reported [24]. In line with our results, a study from New Delhi, India, reported a significant improvement in OGIS in healthy nondiabetic but centrally obese men. The subjects with lower vitamin D levels and greater central adiposity benefited more from vitamin D supplementation concerning improvement in OGIS. No change in HOMA-IR, other fasting insulin sensitivity indices, and lipid profile was observed in the study [7]. A study from eastern India reported that progression to diabetes could be prevented in prediabetes subjects by repletion of low vitamin D levels. The results reflected a decrease in insulin resistance and inflammatory markers TNF-alpha (tumor necrosis factor-alpha) and IL-6 (interleukin-6) associated with it [26]. The improvement in insulin sensitivity/glycemic control by the correction of subnormal vitamin D in prediabetes subjects has been reported by various studies [27-29]. Contrary to our results, Davidson et al. demonstrated no benefit of attaining vitamin D sufficiency on the glucose homeostasis and insulin sensitivity in subjects with prediabetes. They used weekly high dose (mean: 88,865 IU) for one year [30].

The strengths of our study include recruiting prediabetes subjects who fall in a critical zone of suboptimal glucose metabolism spectrum. Also, the analysis of vitamin D levels at baseline and post-intervention was conducted. Furthermore, there was the attainment of vitamin D sufficiency in all subjects who completed vitamin D supplementation. The randomized placebo-controlled design and the study being done in a country which happens to be the diabetes capital of the world and has a high prevalence of hypovitaminosis D add to the significance of the study.

Limitations

There was no estimation of parathormone or serum calcium. The study was powered for OGIS index, and hence sample may not be sufficient to detect changes in other glycemic indices, and, for the same reason, a gender-based analysis was not conducted. Diet, sunlight exposure, physical activity, and other confounders affecting vitamin D metabolism were not taken into consideration; however, randomization may neutralize these limitations. Insulin area under the curve with multiple insulin measurement may better indicate insulin response as the values are dynamic.

## Conclusions

To conclude, the study results and available literature reflect that the OGIS index may be improved in subjects of prediabetes with hypovitaminosis D by correcting the vitamin D levels. The role of vitamin D as a contributor in glucose regulation needs to be explored further to define its role in the management of deranged glucose metabolism. We recommend that subjects with prediabetes should be screened for vitamin D levels and that repletion should be done in subjects with hypovitaminosis D.
